# Evaluation of a Family-Based Intervention Program for Children of Mentally Ill Parents: Study Protocol for a Randomized Controlled Multicenter Trial

**DOI:** 10.3389/fpsyt.2020.561790

**Published:** 2021-01-20

**Authors:** Silke Wiegand-Grefe, Bonnie Filter, Mareike Busmann, Reinhold Kilian, Klaus-Thomas Kronmüller, Martin Lambert, Christine Norra, Kai von Klitzing, Kurt Albermann, Sibylle Maria Winter, Anne Daubmann, Karl Wegscheider, Angela Plass-Christl

**Affiliations:** ^1^Department of Child and Adolescent Psychiatry and Psychotherapy, University Medical Center Hamburg-Eppendorf, Hamburg, Germany; ^2^Section of Health Economics and Health Services Research, Department of Psychiatry and Psychotherapy II, Ulm University, Günzburg, Germany; ^3^Department of General Psychiatry, Landschaftsverband Westfalen-Lippe (LWL) Hospital Gütersloh, Gütersloh, Germany; ^4^Department of Psychiatry and Psychotherapy, University Medical Center Hamburg-Eppendorf, Hamburg, Germany; ^5^Department of General Psychiatry and Psychotherapy, LWL Hospital Paderborn, Paderborn, Germany; ^6^Department of Child and Adolescent Psychiatry, Psychotherapy, and Psychosomatics, University of Leipzig, Leipzig, Germany; ^7^Centre of Social Paediatrics, Cantonal Hospital Winterthur, Winterthur, Switzerland; ^8^Department of Child and Adolescent Psychiatry, Psychosomatics, and Psychotherapy, Charité Universitätsmedizin Berlin, Berlin, Germany; ^9^Department of Medical Biometry and Epidemiology, University Medical Center Hamburg-Eppendorf, Hamburg, Germany; ^10^Department Child and Adolescent Psychiatry, Evangelical Hospital Hamburg Alsterdorf, Hamburg, Germany

**Keywords:** children of mentally ill parents, family intervention, randomized controlled trial, multicenter trial, evaluation

## Abstract

**Background:** Children of mentally ill parents have a three to seven times higher risk of developing mental disorders compared to the general population. For this high-risk group, specialized prevention and intervention programs have already been developed. However, there has been insufficient sytematic evaluation to date. Moreover, effectiveness and the cost-effectiveness data of the respective programs until today is very scarce and at the same time constitutes the pre-condition for the program's implementation into regular health care.

**Methods:** The study consists of a two-group randomized controlled multicenter trial conducted at seven study sites throughout Germany and Switzerland. Participants are families with mentally ill parents and their children aged from 3 to 19 years. The intervention comprises 6 to 8 semi-structured sessions over a period of about 6 months. Topics discussed in the intervention include parental mental illness, coping, family relations and social support. Families in the control condition will receive treatment as usual. The children's mental health, assessed using the K-SADS-PL by blinded external raters will constitute the primary efficacy outcome. Further outcomes will be assessed from the parents' as well as from the children's perspectives. Participants are investigated at baseline, 6, 12, and 18 months after baseline assessment. In addition to the assessment of various psychosocial outcomes, a comprehensive health-economic evaluation will be performed.

**Discussion:** This paper describes the evaluation of a family-based intervention program for children of mentally ill parents (CHIMPs) in the regular health care system in Germany and Switzerland. A methodically sophisticated study design has been developed to reflect the complexity of the actual health care situation. This trial will contribute to the regular health care for the high-risk group of children of mentally ill parents.

**Clinical Trial Registration:** www.ClinicalTrials.gov, identifier NCT02308462; German Clinical Trials Register: DRKS00006806.

## Introduction

Growing up with a parent who suffers from a mental disorder can be a major challenge for the affected children or adolescents. Around the world, 15–23% of all children are assumed to face this challenge (1). In Germany, about 3.6 million children are considered to have at least one parent with a mental illness (2). These children have a 3 to 7 times higher risk to develop a mental disorder compared to the general population. Moreover, the risk for a mental disorder of these children increases with the parental mental stress (3). The costs related to these children's use of mental healthcare services is up to five times higher compared to other children (4). This is why these children are an economically highly relevant target group for the health care system (5, 6).

Both, genetic and environmental factors were identified to account for the association between parents' and children's psychopathology (7–10). The children's mental health problems along with the risk and protective factors associated with the parental disorder can be described on multiple levels, including the child, parental, family, and social level (8).

Previous research has identified numerous risk factors for children's mental health. Risk factors on the parental level are e.g., the specific psychiatric diagnosis, the severity and chronicity of the disease, inappropriate coping strategies and poor emotional availability. On the family level, violence as well as a lack of communication and social isolation are examples of risk factors. The child's temperament and cognitive and social skills, as well as parentification are risk factors on the child level. Regarding the psychosocial level, insufficient social support and a lack of attachment figures outside the family represent risk factors for children's development. These risk factors do not simply add up, but have a multiplying effect (8, 11). Opposite these risk factors, specific protective factors have been identified. These are e.g., primarily positive relationships inside and outside the family, information about the parental disease which is adequate for the children's age and development, adequate individual and familial coping strategies, and supporting parental and family dynamics (12–14).

The tight association of parental mental disorders with the increased risk for mental health problems in children emphasizes the need for early treatment in order to prevent unfavorable development of these children (15–17). Correspondingly, a large number of programs that support families with mentally ill parents has been developed during the last decades. These programs are offered at different levels of the family system: at the child level (e.g., peer support groups, psychoeducational interventions), at the parent level (e.g., parent groups, couple therapy, parent skills groups) and at the family level (e.g., family counseling, family therapy, family assistance) (7, 18, 19).

In respect of intervention programs for children with mentally ill parents, Reupert and colleagues (20) described in their comprehensive review that psychoeducation regarding the parental mental disorder was the key element of the investigated programs. This review also showed that interventions for children of parents with affective or anxiety disorders were overrepresented compared to interventions for other parental diagnoses. Cognitive or behavioral and psychoeducational approaches were the most common therapeutic approaches. Main objects of these approaches are to increase parenting skills and children's knowledge about the parental disease, and to strengthen resilience factors (7). Regarding the programs' effectiveness, Siegenthaler et al. (7) reviewed thirteen randomized controlled trials of preventive interventions for children of mentally ill parents and reported that the risk of developing the same mental illness as the parent was decreased by 40%. Although the effectiveness of prevention and intervention programs was mostly evaluated in randomized controlled trials, the methodical quality of many studies was insufficient (7, 21). The use of cost-effectiveness analyses in the evaluation of these programs was also rare: only one study included an economic evaluation (22). The review by Thanhauser et al. (23) analyzed 50 independent samples from randomized controlled trials, quantifying effects of preventive interventions for children of mentally ill parents. They reported small but significant effects in reducing psychopathology and enhancing mother-infant interaction for different intervention programs. Interventions addressing parents and children jointly produced overall larger effects. The authors state that further studies of high quality are urgently needed. While many programs for the high-risk group of children of mentally ill parents have been developed and evaluated, the implementation of these programs into practice is not yet satisfactory. Even in regions with a high standard of mental health services, affected families do not benefit sufficiently from the respective programs. Assumed reasons are that these programs are not well-established, too expensive and that many parents are afraid of losing the custody for their children if they admit that they need support (24). Moreover, mental health problems are frequently associated with feelings of shame and guilt with the result that often help-seeking is impaired (25).

The cited reviews emphasize the need for further studies with validated outcome measures, high methodic quality, rigorous evaluation designs, high quality cost data and sufficient sample size to implement feasible and acceptable interventions for this risk group (7, 20, 22). A family-based intervention program for children of mentally ill parents is currently implemented and evaluated in a randomized-controlled multicenter trial to address this demand.

The CHIMPs intervention (CHIMPs = Children of mentally ill parents) is based on 4 pillars.

1) The “Model of psychosocial development conditions for children of mentally ill parents” (26) describes the interaction of various risk and resilience factors for children of mentally ill parents.
2) The principles and interventional methods of psychoanalytic family therapy (27).
3) The findings of a needs assessment showed that family-based interventions are well-accepted by families with mentally ill parents, compared to other interventions like for example group interventions (28).
4) The psychoeducational behavior-oriented counseling program of Beardslee et al. (29, 30) is an internationally evaluated approach for families with parental affective disorders. The structure of the CHIMPs sessions is based on the program of Beardslee et al.

Based on these considerations, the CHIMPs intervention was developed in a previous pre- post-trial. The effectiveness of the intervention has been evaluated in a waiting-list control group trial (26). Results of this pilot trial showed that the CHIMPs intervention enhances children's mental health (31), quality of life and social support (32), the parents' coping strategies (33) and family relationships (34).

A crucial element of this study design is the comprehensive evaluation of the cost-effectiveness of the intervention. This trial intends to improve the mental health care situation for children and adolescents affected by a parental mental disorder; first in the participating centers, then nationwide.

## Methods

### Aims and Hypotheses of the Study

The aims of the study are (1) to evaluate the long-term effectiveness of the intervention compared to a control group regarding the children's psychopathology and their health-related quality of life in seven participating sites, (2) to investigate the cost-effectiveness of the intervention in comparison to a control group under conditions of practice. The study for the cost-effectiveness of the intervention is described and published elsewhere (35).

In summary, we assume that the family-based intervention program for children of mentally ill parents will be associated with (1) an improvement of mental health and (2) an improvement of health-related quality of life in children and adolescents of the intervention group compared to the control group after treatment. We expect that these improvements remain stable during the follow-up period.

More specifically, the following hypotheses will be tested:

1) The whole group of children and adolescents (with and without mental health problems) of the intervention group exhibit on average less mental health problems (CBCL) and a higher health-related quality of life (KIDSCREEN) 18 months after baseline assessment compared to the control group.

Those children who have been evaluated having mental health problems at baseline (assessed by CBCL, cut-off T-score > 63) will be analyzed separately regarding the following hypotheses:

2) The proportion of children and adolescents with mental health problems in the intervention group (assessed by CBCL, cut-off T-score > 63) will be lower compared to the control group 18 months after baseline assessment.
3) Children and adolescents with mental health problems of the intervention group will show a better health-related quality of life (KIDSCREEN) 18 months after baseline assessment compared to the children and adolescents with mental health problems of the control group.

Those children who have been evaluated having psychiatric diagnosis at baseline (assessed by K-SADS-PL by an external rater which is blind for the group assignment of the family) will be analyzed separately. To evaluate the effectiveness of the intervention according to this stricter criterion the following primary hypothesis will be analyzed:

4) The proportion of children and adolescents with psychiatric diagnoses (assessed by K-SADS-PL) will be lower in the intervention group, compared to the control group 18 months after baseline assessment.

Additionally, the following secondary hypothesis will be evaluated:

5) Children and adolescents of the intervention group with psychiatric diagnoses will show a better health-related quality of life (KIDSCREEN) 18 months after baseline measurement compared to the children and adolescents with psychiatric diagnoses of the control group.

### Study Design

The present study is a prospective, two-group randomized controlled multicenter trial. In a design with assessments at baseline as well as at 6, 12, and 18 months after baseline assessment, the family-based intervention program CHIMPs is evaluated against a control group. Families in the control group receive treatment as usual (TAU).

Outcomes will be assessed from the patient's, the partner's and the children's perspective (for children aged 10 years and older) as well as from the therapist's perspective. Moreover, the primary outcome of the study (children's mental health) will be externally assessed by rater which is blind for the family's group assignment (K-SADS-PL).

#### Study Centers

The following seven centers, which are located in Germany and Switzerland, are involved in the evaluation of the CHIMPs program: Hamburg (University Medical Center Hamburg-Eppendorf, Germany), Leipzig (University Medical Center Leipzig, Germany), Ulm-Günzburg (Ulm University, Department of Psychiatry and Psychotherapy II, Germany), Wiesbaden-Rheingau (Medical Center Vitos Clinic, Germany), Gütersloh-Paderborn (LWL Community Hospitals, Germany), Berlin (Charité – Universitätsmedizin Berlin, Germany) and Winterthur (Center of Social Pediatrics, Switzerland). All participating study sites will be involved in patient recruitment, diagnostics and in the implementation of the intervention. The study center in Hamburg will furthermore be responsible for the coordination of the study and for the data management. A steering committee at the coordinating site in Hamburg (SW-G, BF, KW) as well as an external scientific advisory board will regularly examine the study's progress. Yearly meetings of the scientific advisory board and monthly meetings of the steering committee will take place. In these meetings, the study progress, the recruitment, the protocol deviations, the loss-to-follow-up, serious adverse events (SAE) and all problems of the study conduct will be discussed.

### Participants

#### Recruitment

Participants will be recruited from in- and outpatient departments of psychiatric clinics in the seven participating study centers. Recruitment will take place at both university and communal hospitals and at child and adolescent as well as adult psychiatric departments. These various access paths reflect the diversity of the population of mentally ill parents and their children.

A member of the research staff will approach the mentally ill parent at the end of his/her treatment period and inform him/her about the project. Moreover, therapists will be asked to inform their patients about the project and to encourage them to contact the study team. After all participating family members provided informed written consent, the study starts with the baseline questionnaires and interviews.

#### Eligibility Criteria

Families with at least 1 mentally ill parent and at least 1 child aged 3–19 years will be included. A parent is defined as mentally ill if he or she has a psychiatric disorder which is treated in an in- or outpatient department at the time of recruitment; in addition, parents currently not in treatment are eligible to participate if they had at least one episode of illness within the last 5 years.

If there is more than one child in the family, families are free to decide with which children they want to participate; however, all family members are encouraged to take part in the study. Couples as well as single parents can take part in the study. Participation is not limited to biological parents; adoptive parents, stepparents as well as foster parents may enter the study.

To compensate for their time conducting the interviews and filling in the questionnaires, each family will receive a staggered financial compensation.

Complete inclusion and exclusion criteria are presented in Table 1.

**Table 1:** Inclusion and exclusion criteria.

**Inclusion criteria^a^**
- Family with at least one mentally ill parent and at least one child between the age of 3 and 19 years -Parental diagnoses: - Mental and behavioral disorders due to psychoactive substance use (F10–F19) - Schizophrenia, schizotypal and delusional disorders (F20–F29) - Mood (affective) disorders (F30–F39) - Neurotic, stress-related and somatoform disorders (F40–F48) - Disorders of adult personality and behavior (F60–F69) - Regular contact between the mentally ill parent and the child/children - Written informed consent with the study protocol - Sufficient knowledge of the German language of parents and children
**Exclusion criteria**
- Severe psychiatric disorders and impairments with acute symptoms such as suicidal tendencies, massive self-injurious behavior, acute psychotic symptoms etc., with indication for inpatient treatment (these patients are placed in inpatient treatment)

a *The numbers in parentheses refer to the codes of the International Statistical Classification of Diseases and Related Health Problems (ICD-10)*.

#### Intervention: CHIMPs

Families in the intervention group will receive the manualized intervention program CHIMPs (26). The psychodynamic CHIMPs approach is based on a theoretical model that addresses coping strategies, family relationships as well as couple and family dynamics. All CHIMPs interventions are derived from these essential issues. Furthermore, the approach is based on key concepts of psychoanalytic family therapy. Moreover, the CHIMPs approach is inspired by Beardslee (29, 36) exclusively in the specific intervention setting of 8 interview sessions with parents, children and the whole family. However, the Beardslee approach is working mainly with cognitive-behavioral psychoeducation. In contrast, the CHIMPs intervention is working consequently with a psychotherapeutic process which is not structured in advance, using the psychodynamical (clarifying, confronting and interpreting) intervention techniques—with the result that the family plays a leading role in the planning of the session content. The psychodynamic approach is also expressed in the multi-generational work at all levels (parents, children, family) that is used for the management of present conflicts. For example, the parents will be asked to tell about how they grew up themselves and which experiences they remember from early childhood in their own family. The work continues with this material. The understanding of the individual psychodynamics of one of the family members will then be worked into the understanding of the psychodynamics of the whole family, and the re-enactment of the primary relationship experiences. Both the work with the parents and with the child is reflecting the respective development experiences. Furthermore, the family dynamic is the object of observation, in so far as it is the representation of earlier relationship experiences and unconscious family conflicts. The relationship dynamics that are developing within the family-therapist-relationship is comprehensible against this background of the reported biographical experiences. Based on these results of the understanding of the dynamics on the different levels we address the focus issues disease coping, family relationships and social network looking at two directions: the reflection of the actual situation as well as entry points for a more positive, intentional, comprehended change process.

Over a period of ~6 months, 8 semi-structured sessions (60–90 min) are conducted with the family. Central themes of the first session are procedure and topics of the CHIMPs program. In the next 2–3 sessions the parental mental illness, the communication and information about the disorder as well as the coping with the disorder within the family are discussed. At the same time, the couple's relationship, the children and the parent-child-relationship are examined; the parental families of origin, the contact with other family attachment figures and the family living conditions are also considered. This information serves as a starting point for the family-based intervention.

Children and adolescents are subsequently invited for one or two individual sessions per child. The main objective is to capture the family situation from the child's perspective with focus on the individual and familial coping as well as relationship structures inside and outside the family.

The core of the CHIMPs intervention is formed by the following three sessions for the whole family that complete the program. All family members together participate in these sessions. Main aim is to reflect the different perspectives that evolved from the single sessions. At the same time, openness, transparency and communication within the whole family are encouraged. The family's present and future handling of the disorder and support from inside and outside the family are reflected on and responded to. Depending on the family's needs and wishes, individual topics like current conflicts can also be discussed. If any family member or the whole family needs further treatment or support, this is initiated by the program staff.

All therapists are experienced in the field of adult or child and adolescent psychiatry or psychotherapy and received a comprehensive 2-day training based on the CHIMPs manual (26). To evaluate adherence to the intervention manual, therapists will complete a self-rate checklist asking for the main topics after each session. In addition, adherence will be evaluated by video or audio recording of sessions and subsequent analysis of the recordings. Furthermore, continuous supervision will enhance adherence to the manual intervention.

The goals of the CHIMPs program can be structured into the three levels family dynamics, coping and relationships. On the level of family dynamics, the main aims are to:

- Link the information on the parental illness to family-historical experiences considering family dynamics
- Promote the understanding of the disorder and the couple and family dynamics from a psychodynamic, multi-generational perspective

On the coping level, the main aims are to:

- Provide both parents with information about the disease (as needed)
- Provide the children with age-appropriate information about the parental mental disorder (as needed)
- Enhance communication about the mental disorder and the problems involved
- Reinforce the coping strategies for the handling of the disorder
- Inform about offers of assistance and encourage greater use of these offers
- Discuss important events maintaining conflicts inside the family, such as hospitalization, loss of employment or change of residence

On the relationship level, the main aims are to:

- Overcome social isolation caused by the parental mental disorder
- Strengthen intra-familial relationships
- Address the topic of extra-familial relationships with the focus on compensating relationships for the child
- Provide knowledge about both risk and protective factors for child development
- Talk about strengths and weaknesses of the child and support options

If participants agree, all sessions are video-recorded for quality control reasons (adherence).

#### Control Group

Families who have been randomly assigned to the control condition will receive treatment as usual (TAU). TAU comprises for example individual psychotherapy for children and/or parents, psychiatric treatment for parents and/or children and support at youth welfare services. Both the control and the intervention group are not prohibited from receiving additional treatment or additional interventions. In the context of the cost-effectiveness analysis, psychosocial and health care service use will be assessed in the intervention and in the control group (35).

#### Sample Size and Power Calculation

The primary analysis is based on the intention-to-treat population of all children and adolescents with initial psychiatric diagnosis (addressed in hypothesis 4). In a first step, we calculated the number of children and adolescents to demonstrate the assumed effect in this population. Since the intervention takes place at family level, we determined the total number of children and adolescents and their families to be recruited in a second step.

We assume that 18 months after the baseline assessment 76% of children and adolescents in the intervention group and 90% in the control group still have a psychiatric diagnosis according to K-SADS-PL. Further, we suppose that 25/55/15/5% of the families have 1/2/3/4 children, so that on average a family has 2 children and adolescents. With a power of 80%, a two-sided alpha of 5% and an intracluster correlation of 0.05, we need 116 children and adolescents per group (232 children and adolescents in total) with an initial psychiatric diagnosis in 58 families per group (116 families in total) to detect this difference. We rounded up to 240 children and adolescents in 120 families. Additionally, we expect that 15% of children and adolescents show an initial psychiatric diagnosis according to K-SADS-PL. With this assumption, we have to recruit in total 800 families with 1,600 children and adolescents.

In this study, we have two additional populations. The first population consists of all children and adolescents (addressed in hypothesis 1) and the second population consists of all children and adolescents with initial mental health problems (addressed in hypothesis 2). For these two hypotheses, we calculated the power with which we can detect the assumed effects based on the sample size calculated for the primary analysis. This sample size enables us to demonstrate with a power of at least 80% the assumed between-groups difference of 0.25 standard deviations in the whole sample (continuous outcomes, hypothesis 1) 18 months after baseline assessment. Furthermore, for the population of hypothesis 2 we expect 400 children and adolescents in 200 families with initial mental health problems if the calculated sample size for the primary hypothesis will be included. In the population, we then can demonstrate a between-groups difference of 10% (80% of the children and adolescents in the intervention group still have mental health problems and 90% in the control group) with a power of 79%.

We used the modules “Tests for Two Proportions in a Cluster-Randomized Design” and “Tests for Two Means in a Cluster-Randomized Design” in PASS 15 (NCSS, LLC. Kaysville, Utah, USA) for these calculations.

#### Outcome Measures

Sociodemographic information including age, gender, number of children, educational and employment status, somatic and psychiatric diseases, history of treatment and current treatment are recorded with a specifically designed questionnaire at baseline.

#### Primary Outcome Measure

Schedule for Affective Disorders and Schizophrenia for School Aged Children, Lifetime Version (K-SADS-PL).

The primary outcome in this study is the children's psychopathology measured by the German version of the K-SADS-PL (37, 38). The K-SADS-PL (Schedule for Affective Disorders and Schizophrenia for School Aged Children, Lifetime Version) is a standardized semi-structured interview for an early diagnosis of mental disorders in children and adolescents according to DSM-III-R and DSM-IV criteria. A trained external rater (blind to the family's group assignment) performs the K-SADS-PL by interviewing one parent for each participating child and adolescent—and in addition all children and adolescents aged 10 years and older about current and past episodes of the children's psychopathology. Children and adolescents are classified as having a psychiatric diagnosis if the interview of either the parent or the child or both indicate a psychiatric diagnosis. The K-SADS-PL has been widely used in research due to its good reliability and validity (39). The K-SADS-PL interviews are audiotaped for quality control.

#### Secondary Outcome Measures

##### Child Behavior Checklist/Youth Self Report

The children's psychopathology will further be assessed by the German version of the Child Behavior Checklist (CBCL) (40) and the Youth Self Report (YSR) (41). Both questionnaires describe a variety of internalizing and externalizing behaviors in children between the ages of 4 and 18. The CBCL consists of 113 items on which the parents are asked to rate the frequency of their children's behavior; a 3-point Likert-scale (0 = does not apply; 2 = clearly/often) is used. In addition, children 10 years of age and older report on their own behavior with the YSR. Children and adolescents are classified as having mental health problems, if the CBCL assessed by a parent or the YSR assessed by the child or both indicate mental health problems (T-score > 63). Both instruments provide raw and T-scores for internalizing and externalizing behavior as well as a total problem score. Due to their satisfactory psychometric properties (42), the CBCL and YSR are among the most widely used questionnaires in research.

##### KIDSCREEN-27

The health-related quality of life of the children is assessed by the KIDSCREEN-27 questionnaire (43). The KIDSCREEN instruments are a set of self-assessment questionnaires used to assess subjective health and well-being of children and adolescents between the ages of 8 and 18; corresponding versions for the parents' assessment of their children's quality of life are available. The short version of the KIDSCREEN questionnaire includes 27 items and covers 5 health-related quality of life dimensions. The questionnaire assesses the child's quality of life in consideration of his or her physical, mental and social well-being. Health-related quality of life is assessed by one parent for each participating child and adolescent and in addition by all children and adolescents aged 10 years and older. A short version of the KIDSCREEN 27 questionnaire, the KIDSCREEN 10 questionnaire, will be used for the calculation of QALYs for children and adolescents (44, 45).

##### Children Global Assessment Scale

In addition, the children's global impairment will be assessed by the German Skala zur Gesamtbeurteilung von Kindern und Jugendlichen (SGKJ) (46), the German version of the Children Global Assessment Scale (CGAS) (47). This scale is the internationally most commonly used scale for the assessment of the severity of psychiatric disorders in children and adolescents. The severity of the disorder is rated on a scale from 1 (needs constant supervision) to 100 (superior functioning in all areas).

##### Brief Symptom Inventory and Patient Health Questionnaire

The parent's symptomatology will be assessed by the German version of the Brief Symptom Inventory (BSI) (48), a short version of the Symptom Checklist-90-Revised (SCL-90-R). This self-assessment questionnaire measures psychological symptoms during the last 7 days. Parents are asked to rate their individual psychological stress on a 5-point Likert-scale from 0 (“not at all”) to 4 (“very strong”). In addition to the BSI, the German version of the Patient Health Questionnaire (PHQ-D) (49) is applied to screen for depressive, anxiety, somatoform, alcohol, and eating disorders. The PHQ-D showed good agreement with the Structured Clinical Interview for DSM-IV (SCID-I) (49); good validity of the PHQ-D for the diagnostic of psychiatric disorders can therefore be assumed. Further aspects of the parental psychopathology will be assessed by the German versions of the Clinical Global Impressions Scale (CGI) (50) and of the Global Assessment of Functioning (GAF) (51). While the CGI is used to measure the severity of the disease, the GAF is used to rate the person's overall level of functioning.

##### European Quality of Life 5 Dimensions Scale

The parents' health-related quality of life is assessed by the German version of the European Quality of Life 5 Dimensions Scale (EQ-5D) (52–54). This health questionnaire expresses the patient's quality of life. The EQ-5D defines the participants' health states at 5 dimensions (mobility; ability to fend for oneself; daily activities; pain; anxiety); The EQ-5D is the most frequently used instrument worldwide for the assessment of health-related quality of life in adults and will be used for the health-economic analyses.

##### Freiburg Questionnaire of Coping With Illness (Freiburger Fragebogen zur Krankheitsverarbeitung)

The Freiburg Questionnaire of Coping with Illness (Freiburger Fragebogen zur Krankheitsverarbeitung, FKV) (55) is a self-report instrument that assesses the parents' coping with the disorder. The FKV assesses a broad spectrum of coping mechanisms on the levels of cognition, emotion and behavior. The short version includes 35 items on the 5-point Likert-scale from 1 (not at all) to 5 (very strong) and is particularly appropriate for follow-up measurements.

##### General Family Questionnaire (Allgemeiner Familienfragebogen)

Family relations and family functioning will be assessed by the German General Family Questionnaire (Allgemeiner Familienfragebogen, FB-A) (56). This self-report instrument focusses on the family system and includes the scales task fulfillment, role behavior, communication, emotionality, affective establishment of relationships, control, values and norms, social desirability, and defense. Forty items are answered on a 4-point Likert-scale ranging from “exact agreement” to “no agreement at all.”

##### Global Assessment of Relational Functioning Scale

In Addition, the dysfunction and relational health of the family is rated based on the German version of the Global Assessment of Relational Functioning Scale (GARF) (51).

##### Oslo Social Support Questionnaire

The Oslo Social Support Questionnaire (OSSQ) (57) measures social support of parents and children. The original version of the OSSQ consists of three items regarding (1) the number of confidants, (2) the sense of interest from other people and (3) the relationship to neighbors. An extended version developed by our research group was used, covering prospective individuals providing social support.

##### Questionnaire for the Assessment of Therapy Outcome (Fragebogen zur Beurteilung der Behandlung)

The evaluation of the treatment will be assessed by the Questionnaire for the Assessment of Therapy Outcome (Fragebogen zur Beurteilung der Behandlung, FBB) (58), which offers the possibility to assess the subjective quality of care according to the two main aspects quality of results (treatment success) and process quality (treatment procedure).

##### Comparative Psychotherapy Process Scales (Vergleichende Psychotherapie Prozess Skalen)

The therapeutic process is also assessed by the Comparative Psychotherapy Process Scales (Vergleichende Psychotherapie Prozess Skalen, VPPS) as the German translation of the Comparative Psychotherapy Process Scale (CPPS) (59, 60). The VPPS was developed to measure therapeutic activity like the process and techniques that are used in the therapeutic setting. It was designed as a primarily descriptive measure (i.e., what was done), but not as an evaluative measure (i.e., how it was done). The aim of the VPPS is to describe the therapeutic session as accurately and objectively as possible.

##### Client Socioeconomic and Services Receipt Inventory

The assessment of psychosocial and health care service use (as a basis for the estimation of treatment costs for the parents) will be performed by the German version of the “Client Socioeconomic and Services Receipt Inventory” (CSSRI-DE) (61). The CSSRI-DE is an interview including five dimensions (sociodemographic data, living conditions, occupation and income, utilization of health services, and medication). The CSSRI-DE was developed for cost analysis of the psychiatric supply system and allows a comprehensive assessment of all substantial components for direct and indirect costs.

##### Children and Adolescent Mental Health Service Inventory

The German version of the “Children and Adolescent Mental Health Services Receipt Inventory” (CAMHSRI) (62) will be used to assess the health and psychosocial service use as a basis for the assessment of treatment costs for the children.

All interviews and questionnaires are administered at baseline as well as at 6, 12, and 18 months after baseline assessment. Although participants are aware of the group they have been assigned to, the external raters assessing the primary outcome and the health-economics, are not.

The measures to be administered at each time point are listed in Table 2.

**Table 2:** Outcomes and measures.

**Outcome**		**Instrument**		**Source**					**Time (months)**			
		**Questionnaire**	**Interview**	**Patient**	**Partner**	**Child (>10 year)**	**Therapist**	**External rater**	**pre**	**6**	**12**	**18**
Demographics		*Ad hoc* items		x	x				x			
Diagnoses/Psychiatric symptomatology	Parents	Medical history						x	x			
		PHQ-D		x	x				x			
		BSI		x	x				x	x	x	x
		CGI						x	x	x	x	x
		GAF						x	x	x	x	x
	Children	CBCL	Kiddie-SADS-PL	x	x				x	x	x	x
		YSR				x			x	x	x	x
		SGKJ						x	x	x	x	x
Health-related quality of life	Parents	EQ-5D		x	x				x	x	x	x
	Children	Kidscreen-27/-10		x	x	x			x	x	x	x
Family relations and family functioning		FB-A		x	x	x			x	x	x	x
		GARF						x	x	x	x	x
		*Ad hoc* items		x	x	x			x	x	x	x
Coping with the disorder	Parents	FKV		x	x				x	x	x	x
Social support		OSSQ		x	x	x			x	x	x	x
Evaluation of treatment		FBB^*^		x	x	x	x			x	x	x
Health economic assessment of treatment costs	Parents		CSSRI-DE	x	x			x	x	x	x	x
	Children		CAMHSRI-DE	x	x			x	x	x	x	x
Formulation of goals		*Ad hoc* items^*^		x	x	x			x			
Achievement of goals, satisfaction		*Ad hoc* items^*^		x	x	x	x			x	x	x
Therapeutic process		VPPS^*^					x		x^**^			
		*Ad hoc* items^*^					x		x^**^			

* *For intervention group only*.

** *During the intervention*.

#### Randomization Procedure

Computerized lists for familywise randomization with an allocation ratio of 1:1 stratified by centers with a variable block length will be prepared by the Department of Medical Biometry and Epidemiology of the University Medical Center Hamburg-Eppendorf. The subsequent central allocation procedure is managed by the coordinating study center in Hamburg to guarantee allocation concealment.

Randomization will take place once the standardized baseline diagnostics are complete. Thus, every randomized family, both in the intervention and in the control group, will receive comprehensive standardized diagnostics of every participating child.

#### Data Assessment and Data Management

**Figure 1:**
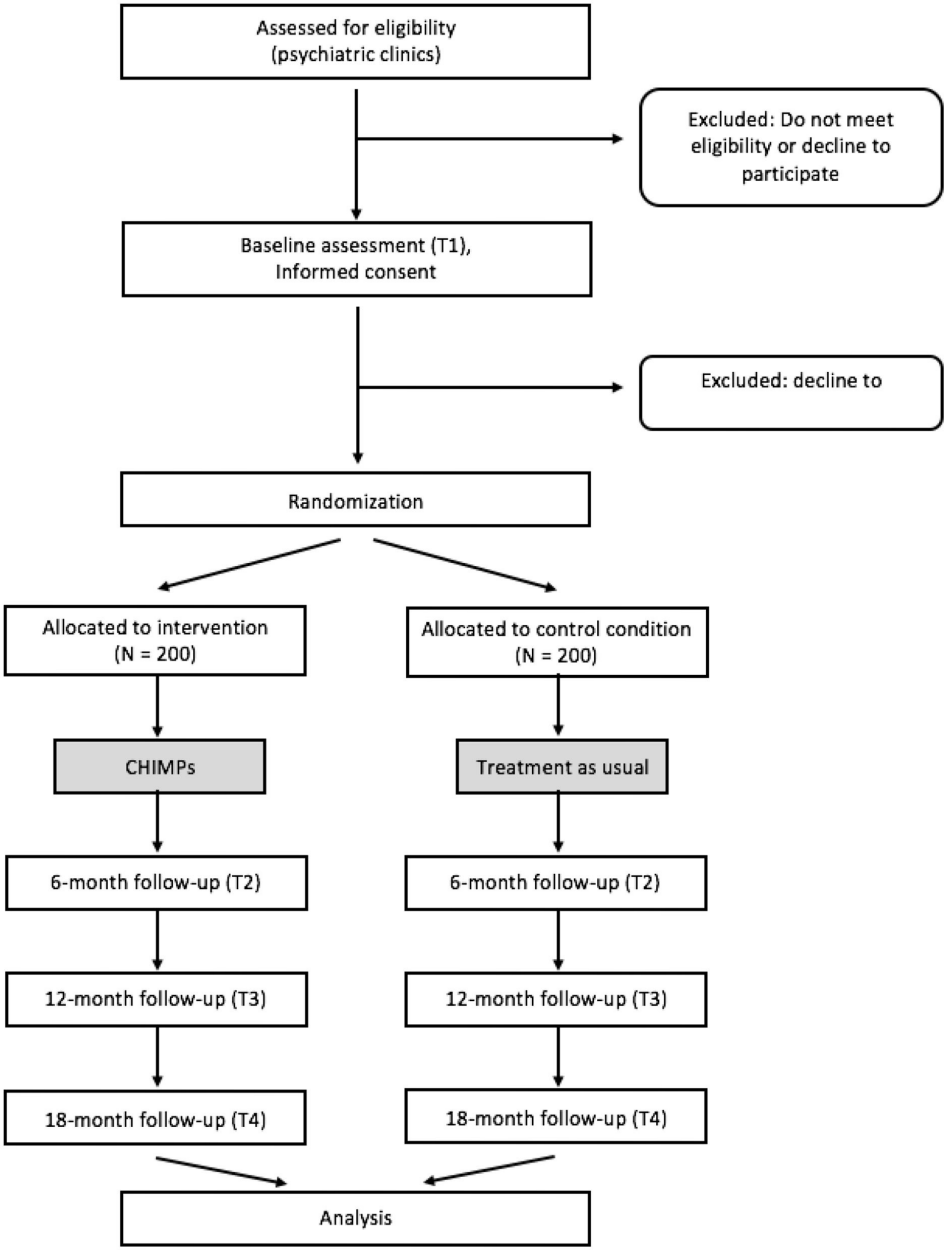
Overview of the CHIMPs study design.

At the beginning of the study (T1; see Figure 1), the mentally ill parent and—if there is one—the partner fill in the baseline questionnaires and participate in the K-SADS-PL interviews about their child/children. All children aged 10 years and older are also asked to complete this diagnostic process. As soon as all documents are completed, the family is randomized to either control or intervention group. The research staff subsequently informs the family about their group assignment and provides feedback on the results of the child's K-SADS-PL interview.

If the family has been referred to the intervention group, the therapist who carries out the CHIMPs program is informed and the start of the intervention is initiated. If the family has been referred to the control group, no further treatment is provided within the CHIMPs study.

At 6 (T2), 12 (T3), and 18 (T4) months after baseline assessment, the families will be contacted again by the research staff. The questionnaires and diagnostic interviews for all three follow-up assessments are the same as at the baseline assessment. Families receive a financial compensation (up to €50) if a follow-up assessment is completed, in order to further promote a complete follow-up.

All study-related information will be stored securely at the study site. All records that contain names or other personal identifiers, such as locator forms and informed consent forms, will be stored separately from study records identified by code number. The coordinating study site will oversee the intra-study data sharing process.

All study outcomes, especially the primary outcome papers, will be published in peer-reviewed journals and presented at scientific conferences. All papers and abstracts must be approved by the coordinating site before submission.

### Data Analysis

All data will be entered twice at the coordinating site. Original study forms will be sent to the coordinating site and a copy of them will be kept at the participating site. Participant files will be stored in a secure and accessible place for a period of 3 years after completion of the study.

Data will be analyzed and reported according to the CONSORT statement. Mean and standard deviation will be presented for the continuous variables and absolute and relative frequencies for the categorical variables for the whole sample and separated by treatment groups. Since the relatives of a family are subject to common influences, cluster effects are generally to be expected. Therefore, the inferential statistics will be conducted using mixed models with the family and the relatives as random effects. As further covariates, recruitment center, treatment group, time, and the interaction between the two, as well as baseline variables that are unbalanced between the treatment groups. As further possible covariate, the type of clinic (university clinic vs. supply clinic) will be examined. For this purpose, baseline comparisons will be performed. If significant differences are found, this variable will be included in the model as a further fixed effect. We will use the direct Maximum-Likelihood as the statistical estimation procedure in the intention-to-treat population (ITT), which results in unbiased estimations under the missing-at-random assumption. The ITT analysis will be based on the available clinical data from all randomized families. The primary outcome analysis is the treatment group contrast (CHIMPs vs. TAU) at 18 months after baseline assessment in the ITT population of the children with an initial psychiatric diagnosis according to K-SADS-PL in a mixed logistic regression with the above-mentioned specification. The primary outcome is a psychiatric diagnosis according to K-SADS-PL (yes/no). Only the result of this comparison will be interpreted in a confirmatory manner. Additionally, this analysis will be repeated in the per protocol population. Sensitivity analyses will be performed for the primary outcome analysis with different methods of missing value imputation such as multiple imputation and last observation carried forward (LOCF) to study the robustness of the findings. Odds ratios, their 95% confidence intervals and *p*-values will be reported. The type I error will be set at 5% (two-sided). The secondary outcomes will be examined with appropriate methods in the ITT population in an exploratory manner. As far as possible, these outcomes will be operationalised as difference to baseline. Interim analyses are not planned. Subgroup analysis will be conducted on study sites, children's and adolescents' age and children's and adolescents' gender. A detailed statistical analysis plan will be finalized based on an analysis of a blinded, pooled data set. Statistical analyses will be carried out with SPSS, version 24 or newer (IBM Corp, Armonk, NY, USA) R, version 3.6.3 or newer (R Foundation for Statistical Computing, Vienna, Austria) or SAS, version 9.4 or newer (SAS Institute, Cary, NC, USA).

Cost differences between both study groups will be analyzed by linear regression models (63–65). The adjustment of standard errors in case of deviations from the normal distribution will be performed by non-parametric bootstrapping (65, 66). The cost-effectiveness of the CHIMPs intervention compared to treatment as usual (TAU) will be computed by the net-monetary-benefit method (67–69). Quality adjusted life years (QALYs) will be computed by transforming KIDSCREEN-10 values into Child Health Utility (CHU9D) values (70) using the algorithm provided by Chen and colleagues (71). Incremental cost-effectiveness ratios (ICER) will be calculated to estimate the maximum willingness to pay necessary for the gain of one quality-adjusted life year (QALY) by implementing the CHIMPs intervention in comparison to standard care (69). ICER will be interpreted on the basis of the cost-effectiveness plane (67–69). Stochastic uncertainty of the ICER will be estimated by non-parametric bootstrapping and the cost-effectiveness acceptability curve (67–69).

## Discussion

The present study will implement an intervention program for families with mentally ill parents in regular care at multiple sites in Germany and at one site in Switzerland. In the current trial, the program's effectiveness and cost-effectiveness will be evaluated in a randomized-controlled design with a comprehensive health-economic assessment at baseline, 6, 12, and 18 months after baseline assessment. We hypothesize that the CHIMPs intervention improves children's mental health and health-related quality of life compared to the control condition. An important strength of this study is the use of a randomized controlled trial design with a control group receiving treatment as usual. The study design includes a comprehensive health-economic evaluation.

The low-restrictive inclusion criteria allow patients with a variety of psychiatric disorders to take part in the study. Moreover, not only biological parents, but also adoptive parents, foster parents or step parents will be included. This represents an important opportunity to investigate a sample that is representative for this diverse high-risk population in a standard environment.

The CHIMPs family intervention of the present study involves all family members. This setting of the intervention represents an important strength of the study as existing research proves that addressing parents and children jointly produced larger effects of preventive interventions for children of mentally ill parents compared to interventions addressing parents and children individually (23).

In the CHIMPs study, psychopathology will be assessed by multiple perspectives. Most importantly, children's mental health, the primary outcome, will be assessed by a clinical interview by an external rater blind to the family's group assignment. Moreover, psychopathology of the children will be measured by parents' and children's perspectives using the CBCL and the YSR for children 10 years and older. Overall, a wide range of outcome measures (e.g., mental health, health-related quality of life, coping, social support, family relationships) for children and parents will be assessed from multiple perspectives. As multiple perspectives are estimated to be state-of-the art measuring psychopathology (72, 73) and quality of life (74) in children and adolescents, this assessment presents another clear strength of our study.

The extensive assessment of outcomes will allow for a comprehensive analysis of various risk and protective factors relevant for the interplay between parental mental disorders and children's mental health. This analysis will facilitate the further understanding of transgenerational transmission of mental disorders and the adaptation of existing support programs for families with parental mental health problems. Moreover, this study aims at reducing transgenerational transmission of mental health problems in families with parental mental disorders.

An important implication for practice is that all children participating in the study are screened for mental health problems at the very beginning of study participation. This provides the opportunity for an early identification of children with mental health problems who will subsequently be referred to appropriate offers of assistance. All children and adolescents participating in the intervention or the control group with indication for individual support like e.g., psychiatric treatment or psychotherapy will obtain the corresponding referral.

Difficulties could arise from the multicenter design of this study. The combination of both university and supply clinics could lead to differences in the recruitment process. For this reason, possible center effects will be analyzed and treated accordingly during the statistical analysis.

As a large number of therapists from different therapeutic approaches will lead the CHIMPs program, it could be difficult to perform the intervention in a reliable standardized manner. To ensure adherence to the manual, the process and the methods of each session will be documented and analyzed for compliance with the manual.

Experience from this trial will be used to revise the existing manual. The financing of the intervention in regular care beyond the duration of the study will be discussed with health-insurance representatives as part of the study. An important aim of the study is to not only evaluate the CHIMPs intervention in the participating study centers, but to implement it into regular care throughout Germany.

The support provided to families with mentally ill parents should be comprehensive and integrative and therefore oriented toward all family members as the psychosocial and medical starting points for the affected families are equally complex. If successful, the CHIMPs intervention could be the first program for mentally ill parents and their children to be implemented nationwide into regular practice; this could be an important and future-oriented contribution to the improvement of a family-oriented support system for this particular risk group.

## Ethics Statement

The studies involving human participants were reviewed and approved by the Hamburg Medical Council as well as by the Ethics Committees of all participating study sites. Written informed consent to participate in this study was provided by the participants' legal guardian/next of kin.

## Author's Note

The full trial protocol can be accessed at Silke Wiegand-Grefe, Department of Child and Adolescent Psychiatry and Psychotherapy, University Medical Center Hamburg-Eppendorf, Hamburg, Germany.

## Author Contributions

SW-G is the principal researcher of this project. SW-G and AP-C have developed and evaluated the CHIMPs intervention. AP-C, SW-G, and BF have drafted the manuscript. BF and MB are responsible for the data and study management in the coordination study center. SW-G, RK, K-TK, KK, KW, and ML were significantly involved in the conception of the study and participated in its design with study advisory by AP-C. CN, KA, and SW are responsible for the realization of the project at their study sites. AD and KW wrote the paragraph on statistical methods. SW-G, BF, KW, and MB are responsible for the data and study management. All authors contributed to the editing of the manuscript and have read and approved the final manuscript.

## Conflict of Interest

The authors declare that the research was conducted in the absence of any commercial or financial relationships that could be construed as a potential conflict of interest.
